# Microbial short-chain fatty acids: a bridge between dietary fibers and poultry gut health — A review

**DOI:** 10.5713/ab.21.0562

**Published:** 2022-04-30

**Authors:** Qasim Ali, Sen Ma, Shaokai La, Zhiguo Guo, Boshuai Liu, Zimin Gao, Umar Farooq, Zhichang Wang, Xiaoyan Zhu, Yalei Cui, Defeng Li, Yinghua Shi

**Affiliations:** 1 Department of Animal Nutrition and Feed Science, College of Animal Science and Technology, Henan Agricultural University, Zhengzhou, Henan, 450002, China; 2 Henan Key Laboratory of Innovation and Utilization of Grassland Resources, Zhengzhou, Henan, 450002, China; 3 Henan Herbage Engineering Technology Research Center, Zhengzhou, Henan, 450002, China; 4 Department of Poultry Science, University of Agriculture Faisalabad, Sub Campus Toba Tek Singh 36050, Pakistan

**Keywords:** Dietary Fibers, G-protein-coupled Receptors, Gut Microbiota, Histone Deacetylases, Short-chain Fatty Acids

## Abstract

The maintenance of poultry gut health is complex depending on the intricate balance among diet, the commensal microbiota, and the mucosa, including the gut epithelium and the superimposing mucus layer. Changes in microflora composition and abundance can confer beneficial or detrimental effects on fowl. Antibiotics have devastating impacts on altering the landscape of gut microbiota, which further leads to antibiotic resistance or spread the pathogenic populations. By eliciting the landscape of gut microbiota, strategies should be made to break down the regulatory signals of pathogenic bacteria. The optional strategy of conferring dietary fibers (DFs) can be used to counterbalance the gut microbiota. DFs are the non-starch carbohydrates indigestible by host endogenous enzymes but can be fermented by symbiotic microbiota to produce short-chain fatty acids (SCFAs). This is one of the primary modes through which the gut microbiota interacts and communicate with the host. The majority of SCFAs are produced in the large intestine (particularly in the caecum), where they are taken up by the enterocytes or transported through portal vein circulation into the bloodstream. Recent shreds of evidence have elucidated that SCFAs affect the gut and modulate the tissues and organs either by activating G-protein-coupled receptors or affecting epigenetic modifications in the genome through inducing histone acetylase activities and inhibiting histone deacetylases. Thus, in this way, SCFAs vastly influence poultry health by promoting energy regulation, mucosal integrity, immune homeostasis, and immune maturation. In this review article, we will focus on DFs, which directly interact with gut microbes and lead to the production of SCFAs. Further, we will discuss the current molecular mechanisms of how SCFAs are generated, transported, and modulated the pro-and anti-inflammatory immune responses against pathogens and host physiology and gut health.

## INTRODUCTION

The development of the gastrointestinal tract (GIT) starts at an early embryonic stage where certain factors including dam, diet, and environment affect its formation. During embryonic development, several species of microflora from the hen’s reproductive tract and eggshell moved to the embryo, and soon after birth, they start multiplying rapidly while certain other bacterial species and microorganisms from eggshells, environment, and feed also join existing microbiota and start colonization in certain parts of GIT [1]. Among the bacterial species, many acts as beneficial for the host and play important role in several physiological processes such as nutrient absorption, metabolism, tissue development, immune homeostasis, and maintaining overall health [2]. Microflora regulates these processes by the production of different metabolites such as short-chain fatty acids (SCFAs) which are mainly produced by colonic anaerobic bacterial fermentation of dietary fibers (DFs) [3]. The SCFAs are chemically composed of hydrocarbon chains and carboxylic acid moiety [4]. In poultry, the most frequently studied SCFAs are the acetate, propionate, and butyrate with two, three, and four carbon molecules in their chemical structure while their effect on several systems has been accentuated both at cellular and molecular levels [5].

In the past two decades, there has been immense research on optimizing poultry gut health by modulating intestinal microbiota through dietary interventions, and in this regard, DFs seemed important [6]. There is a difference between single and compound stomach animal species for the digestion of fibers. Unlike compound stomach animals, the microbial fermentation of DFs in single stomach animals like chicken mainly takes place in the hindgut though each part of GIT harbors its own specific microbiota. The DFs greatly affect the poultry’s gut physiology by modulating its microbiota and eliminating solid waste products [7]. Previously localization of different microbiome in GIT has been described in chicken [8] however the interaction between DFs and gut microbiota has received little attention [9]. Similarly, there is also a need to focus on interaction between DFs, mucus, and epithelial cells which play a central role in development of physicochemical and immunological barriers to restrain the microbiota and to halt invading pathogens, antigens, microbial populations, and toxins [10].

In this review, we have focused on clear and recent narrations of the microbially-derived SCFAs on host tissues as well as their cellular and molecular mechanisms. For this, we have mainly focused on different sources of DFs, their interrelationship with microbiota, and their influence on mucus. For better understanding, we have summarized the intestinal epithelial cell (IEC) polarity, generation mechanisms, and transportation of microbially-derived SCFAs, and their role in modulating pathophysiological changes in the gut, histone deacetylases (HDACs) inhibition, and pro-and anti-inflammatory consequences on immune cells.

## TYPES OF DIETARY FIBERS AND THEIR IMPACT ON GASTROINTESTINAL TRACT

Dietary fibers are homogenous or heterogenous carbohydrate polymers with three or more monomeric units. They are resilient to digestion by host endogenous enzymes and belong to the following three categories: i) present naturally in the foods and available to the farm animals (i.e. cereals, legumes, etc.), ii) acquired from raw food materials via chemical, enzymatic, and physical means and iii) synthetic ones such as polydextrose. The DFs can also be defined based on their chemical composition and nutritional functions such that chemically they are the sum of lignin and non-starch polysaccharides while nutritionally they are the carbohydrates indigestible by host endogenous enzymes [11]. DFs are also categorized depending upon their primary food sources, water-solubility, chemical structure, and degree of fermentation. They are subdivided into resistant starch (RS), resistant oligosaccharides, polysaccharides, soluble and insoluble forms [12]. All types of fiber, however, do not exist in the same category of food such as RS only exists in cereals and legumes, while arabinoxylans and β-glucans are found only in cereals [13]. In the upper part of the GIT, RS withstands enzymatic digestion in pigs, so their digestion takes place in the hindgut where they yield butyrate-producing *Bifidobacterium adolescentis*, *Parabacteroides distasonis*, *Faecalibacterium prausnitzii*, and total SCFAs [14].

The most useful sources of DFs that modulate the poultry gut microbiota and contribute to poultry health are given in Table 1. Inulin supplementation had been shown to improve host gut inflammatory responses and gut health through promoting *Lactobacilli* and *Bifidobacteria* colonization and increasing SCFAs production largely butyrate, acetate, and propionate in broilers which exert antibacterial properties to combat *Salmonella* infection in chicks [15]. In addition, the intake of dietary fructooligosaccharide enhances the production of SCFAs and reduces the colonization of *Salmonella* spp., *Clostridia perfringens*, and *Escherichia coli* in broiler chickens [16]. Similarly, supplementation of cellulose affects the composition of microbiota at a level of phylum *Bacteroidetes* and genus *Alistipes* in the cecum of Saanen goats [17], whereas this genus may improve the performance of broilers by producing succinate as an end product [18]. Arabinoxylan utilization by ducks had increased the stimulation of *Megamonas* and *Bifidobacterium* spp., which further resulted in increased concentration of SCFAs (butyrate, acetate, and propionate) and branch-chain fatty acids (isobutyric acids) [19]. The fermentation of DFs in poultry depends on their physico-chemical properties and the matrix. Most of the DFs such as pectin does not cause fecal bulking effect and are gradually fermented by the gut microbes to produce SCFAs however, several insoluble forms such as cellulose, hemicellulose, and lignin cause fecal bulking effects and are either partly digested by the gut microbes in intestine or excreted as such. Similarly, some of the soluble non-starch polysaccharides polymers with high molecular weight (i.e. β-glucans, guar gum, psyllium, and pectin) are viscid and form a gel-like structure in the intestinal tract and influence the postprandial metabolism of lipids and delay glucose absorption in humans and pigs [12].

It is suggested that young birds should be fed on low levels of DFs (less than 1.5%) diets because high levels decrease nutrient digestibility in early life period and increase transit speed of digesta [20]. During growing phase, however, inclusion levels of 2% to 3% DFs are recommended which improve the gizzard size and feed efficiency in poultry species [21]. For example, the quails fed on a 1.5% wheat bran-based diet showed increased relative length of intestinal segments, villi to crypt depth, villi height, and villi thickness [22]. Whereas lignin supplementation in geese decreased villi length but on the contrary, the supplementation of pectin, alfalfa, and rice hulls had a positive effect on villi height [23,24]. Efforts are on to increase fiber inclusion rate in poultry feed by fortification with the extracted non-digestible carbohydrates. A large variety of fortified oligosaccharides and carbohydrate polymers are commercially available in the form of prebiotics which increases the population of beneficial bacteria in the gut [25]. In addition to their natural presence in different foods, DFs-rich ingredients or isolated DFs molecules can be added to the diet by technological means to provide benefits for extra health.

## INTERRELATIONSHIP OF MICROBIOTA, DIETARY FIBERS, AND DIGESTION

Feed ingredients differentially affect the bacterial communities and the production of metabolites depending on their particle size, type, and chemical properties. The DFs act as a carrier of feed anti-oxidants (AOXs). Though not reported in chickens yet, the main physiological function of DFs is to convey AOXs across the GIT. Upon reaching the colon, AOXs secrete a fiber matrix to produce an AOX environment and reveal metabolites [26]. The type of fiber reaching the posterior gut is the key in defining the type of bacteria and the metabolites (SCFAs) being produced. Such that supplementation of pigs diet with RS increased SCFAs production by 34% compared with the digestible starch diet [27]. Though corn-soybean meal-based diet increases the concentration of *Lactobacillus* spp. and SCFAs production in duodenum, jejunum, and ileum in broilers of all age groups yet high DFs diets based on corn-soybean meal-dried distillers grains and wheat bran produce even higher SCFAs compared with low DFs diets (i.e. corn-soybean meal) [28]. So increased production of SCFAs by dietary manipulation with DFs reflects that fiber maintains integrity and diversity of the GIT microbiota which further increases the fermentation of complex fibers and release of energy for the host. Chickens, ducks, and geese do not produce enzymes as hosts to break down DFs such as fructooligosaccharides, xylooligosaccharides, and mannan [29]. In these species, DFs are believed to reach the ceca in undigested form and then undergo microbial fermentation. Culture-based studies showed that different bacterial genera i.e. *Clostridium*, *Bacteroides*, and *Bacillus* synthesize mannanases that can unfold *β*-1,4 mannopyranoside bonds in mannan products [29]. Metagenomics analysis of ceca in chickens showed over 200 non-starch polysaccharides degrading enzymes, oligosaccharide- and polysaccharide-degrading enzymes, and several pathways related to SCFAs production which highlight the functional properties of the cecal microbiota [30]. At present exogenous enzymes including phytases and carbohydrases such as amylases, xylanases, and β-glucanases are used in broilers to degrade complex carbohydrates into their respective sugars or amino acid components [31]. Bacteria can then ferment them into metabolites (i.e. SCFAs), CO_2_, H_2_, branched-chain fatty acids, ammonia, and other carboxylic acids [32]. However, to reduce the use of exogenous enzymes, future studies need to focus on identifying specific intestinal bacteria that promote different enzymatic reactions to support the fermentation of DFs in poultry species.

## INTERACTION OF DIETARY FIBERS WITH GUT MUCUS

The entire surface of the chicken GIT is protected by a layer of mucus. Mucus is a glycoprotein and being a part of innate host, immune response is produced by goblet cells. It protects the IECs from bacterial, mechanical, and chemical injuries [33]. It halts the direct interaction of luminal antigens with the epithelium. The DFs have a significant effect on mucus secretion, cell proliferation, and changing luminal environment thus they are involved directly or indirectly with intestinal health [34]. To maintain the mucus layer, however, the genetic makeup of the host and interaction of intestinal microbiota with DFs are important. The goblet cell numbers are used as an indicator of mucus production in monogastric animals [35]. In golden hamsters feeding different sources of DFs i.e. oat bran and rye bran were found to improve goblet cell numbers in the gut, while pectin was also found to intensify the mucus layer via a high-water holding capacity mechanism (dehydration combat mechanism) [36]. Therefore, they are known as a stimulator of goblet cells proliferation. The role of DFs in increasing the *mucin2* gene expression, fecal mucin output, and goblet cell numbers has also been reported recently [37]. A lower level of DFs predisposes redundancy of mucus layer and can lead to infectious susceptibility and the reclamation of chronic inflammatory diseases [38]. A higher level of DFs however, can cause loss of endogenous amino acids. For example, DFs have been found to increase the number of mucin-producing goblet cells. Mucus contains a high concentration of mucin (a threonine-rich glycoprotein) that becomes scarce during high mucus production [39].

The IECs are enclosed by a mucus layer, thus keeping the bacteria away from mucosa deterioration [40]. Intestinal microbiota ferments DFs and produces SCFAs and both stimulate mucus production and regulation. The *Bacteroides thetaiotaomicron* spp. which produces acetate and propionate, also stimulate goblet cell differentiation and expression of mucin-related genes. Moreover, *Faecalibacterium prausnitzii* spp. uses acetate and can produce butyrate, thus stabilizing the proper physiology of the gut epithelium by averting the overproduction of mucus.

## MECHANISMS TO CONTROL INTESTINAL EPITHELIAL CELL POLARITY, ROLE OF DIETARY FIBERS, AND GUT HEALTH

### Introduction of intestinal epithelial cell polarity in intestine

**Figure 1:**
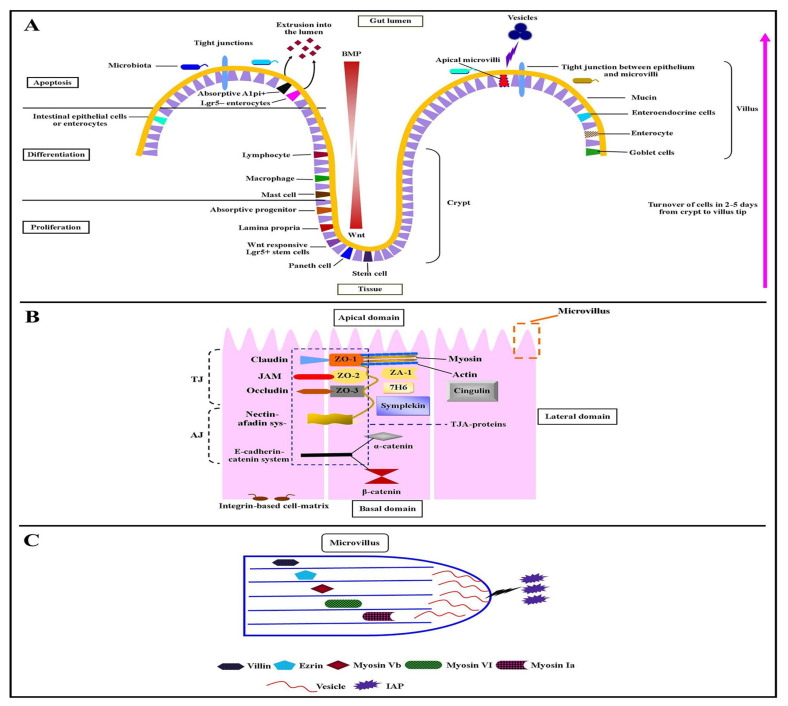
A depiction of epithelial cell polarity. (A) Turnover of cells in 2–5 days from crypt to villus tip and (B) α-catenin in Figure 1. (C) The box area from panel B describes the single microvillus within its protein components such as villin, ezrin, myosin Vb, myosin-VI, and myosin-Ia, and the microvillus-derived vesicle with alkaline phosphatase enzyme.

The mammalian GIT consists of distinct layers of cells. It requires precise interaction between each layer or cell type to perform different functions i.e. absorption of dietary nutrients and water, discharge of waste products via peristaltic contractile movements, and maintaining a physical barrier against pathogens. The two most remarkable cell populations of the intestine are the muscle cells and epithelial cells. The intestinal epithelium is distributed into two parts; flask-shaped submucosal invaginations- crypts, and finger-like luminal protrusions- villi [41]. The IECs start their growth from Wnt responsive Lgr5^+^ stem cells (multipotent stem cells), differentiate, and get mature at the base of crypts [42]. While the absorptive Alpi^+^, Lgr5^−^ enterocytes move up toward the villus until they reach the villus tip where they go through apoptosis and shed themselves into the lumen to balance defensive barrier under different physiological conditions [43] (Figure 1). This process of turnover lasts for two to five days followed by a bone morphogenetic protein (BMP) signaling pathway [44].

IECs are organized as a monolayer of columnar-shaped, polarized epithelial cells (Figure 1A). The surface of IECs is divided into two domains; apical domain—facing to the lumen of the gut, and basolateral domain—facing to the intestinal tissue. The basolateral cell-surface domain is further divided into a basal (face basement membrane) and a lateral domain (face neighboring cells) (Figure 1B). The basal surface domain is a home for integrin-based cell-matrix adhesions, through which the cells are interconnected with the basement membrane. While the lateral surface domain is a house for intercellular adhesions such as E-cadherin-based adherens junctions and claudin-based tight junctions (TJs) [45] (Figure 1B). The intestinal tight junction-complex-associated proteins consist of surface membrane proteins (i.e. claudin, occludin, junctional adhesion molecules, the coxsackievirus and adenovirus receptors proteins), and intracellular proteins (i.e. zona occludens [*ZO-1*, *ZO-2*, and *ZO-3*], cingulin, *7H6*, symplekin, and *ZA-1*) [46]. It has been documented that the TJs and polarized intracellular trafficking machinery are the central players in the IECs’ polarity and establish communications between gut lumen and bodily tissues to maintain immune tolerance with commensal bacteria and control gastrointestinal pathogens [47]. The adherens junction consists of the E-cadherin-catenin system and nectin-afadin system. The partner of nectin that is known as “afadin” directly or indirectly binds with a number of proteins, including zona occludens proteins. Several studies demonstrated that the modulation of selectively induced *Bifidobacterium* spp. enhanced barrier function and the function of tight junction-associated proteins [48]. The TJs have further joined with the actin filament-based projections of apical plasma membrane known as microvilli (Figure 1B).

### Role of apical plasma membrane microvilli and their protein components in intestinal epithelial cell polarity

The apical plasma membrane microvilli and their protein components such as villin, ezrin, myosin-Vb, myosin-VI, and myosin-Ia play important role in IEC’s polarity. Villin is an actin-modifying protein situated in the basic subapical terminal web and the apical plasma membrane microvilli (Figure 1C) [49]. Villin expression in IECs represents induced inflammation and related lesions under inflammatory bowel disease, which highlights the dynamics of IEC’s polarity in gut wound healing, immunopathology, and intestinal epithelial homeostasis. In humans, the depletion of myosin-Vb protein in microvillus due to disease and *MYO5B* mutations results in inactivation of ezrin protein at apical surface and cause microvillus atrophy in the IECs [50]. The loss of another component of microvilli i.e. myosin-Ia results in tumor development, loss of IECs polarity, and carcinogenesis in mice [51]. The function of myosin-VI and myosin-Ia controls the base-directed movement and microvillus tip of plasma membrane respectively [52]. Both of these myosins regulate the circulation of brush-border enzymes such as intestinal alkaline phosphatase (IAP) and intramicrovillus (microvillus tip and microvillus base). The tips of the apical microvilli produce vesicles that are extruded into the gut lumen [53]. The microvillus-derived vesicles are rich in IAP (Figure 1C). The overexpression of this enzyme under the exposure of IECs to *E. coli* gives rise to an increased abundance of microvilli-derived vesicles [54].

### Role of dietary fibers in controlling intestinal epithelial cell polarity

The DFs such as wheat bran has been used in controlling colonic mucosal proliferation and in preventing proliferative diseases of colon [55]. Thought, specific therapeutic strategies such as DFs, probiotics, and prebiotics supplementation have been used to manage some of the diseases including obesity, type-1 diabetes, and colorectal cancer in mice, and different pathogens causing diseases such as *salmonella* enteritidis, necrotic enteritis, and *clostridium perfringens* in broilers [56]. Up to date in poultry, it is not clear yet whether the DFs, probiotics, prebiotics, and the microbial-derived metabolites (i.e. SCFAs) affect polarized intracellular trafficking machinery involving proteins such as villin, ezrin, myosin-Vb, myosin-VI, myosin Ia, and IAP and either their (proteins) expression increase the stimulation of microvilli-derived vesicles, establishing intestinal epithelial homeostasis, wound healing, and tumor suppression or not. There is a need to get insight into the IEC polarity to understand the mechanisms involved in the maintenance of epithelial homeostasis, immune system by commensal bacteria, and balanced communications among gut lumen, body tissues, and gastrointestinal pathogens. Owing to fast differentiation and turnover the IECs have become a focus of the current research. However various factors such as diets, diseases, hormones, and genetics affect IECs turnover and differentiation along crypt-villus axis.

## INTERPLAY BETWEEN DIETARY FIBERS AND MICROBIOME GIVES RISE TO METABOLITES (SHORT-CHAIN FATTY ACIDS)

Metabolites are small molecules produced by metabolic reactions, catalyzed by gut enzymes or bacterial fermentation of various foods. Through these small molecules, the gut microbiota communicates and makes a tensile network with the host [57]. The microbial metabolites affect host immune maturation, energy metabolism, immune homeostasis, and mucosal integrity. Variation in the gut microbial metabolites has been illustrated in many studies during salmonellosis, *E. coli* infection, necrotic enteritis condition, and *Campylobacter jejuni* infection [58]. Beneficial microbiota produces SCFAs including acetic acid, propionic acid, and butyric acid which have bacteriostatic ability to destroy campylobacteriosis, salmonellosis, and *E. coli* causing bacteria.

In this context, the composition and particle size of diet not only maintain host health but also modulates the beneficial microbial communities, their richness, and diversity in the digestive tract. The origin, type, and quality of diet modulate the gut microbiota in a time-dependent manner. Long-term dietary regimes particularly plant-based and animal fat or protein-based diets are associated with so-called enterotypes. This dichotomy in the plant-based diet/animal-based diet ratio was also observed in broilers and laying hens arguing that these bacterial communities are driven by a long-term modification in the diet [59].

Gut microbiota can ferment undigested carbohydrates in most parts of the GIT, but in poultry, this process mainly occurs in crop and caecum where bacteria are abundantly populated. The crop and ileum are the main lactic acid-producing repertoires, owing to the presence of a high concentration of *Lactobacillus* spp., as opposed to caeca where the quantity of butyric, acetic, and propionic acids are higher [60]. Furthermore, Jozefiak et al [6] observed that the cereal-type feed ingredients such as wheat, rye, and triticale determine the quantity of acetic acid in the caecum except for the crop and gizzard. This suggests that the application of DFs is a potential way to influence SCFAs concentration. The SCFAs enhance beneficial microbial populations to regulate endogenous enzymatic activities and produce more energy and carbon for IECs [61], thus contributing to maintaining mucosal integrity, immunity, and health of broilers, geese, and ducks [3]. Therefore it is important to highlight the potential role of specific types and sources of DFs that can be used in manipulating the commensal bacterial populations which specifically induce the synthesis of SCFAs. Similar studies in poultry species are warranted to investigate possible involvement of DFs in early postnatal development and microbially modulated DNA methylation.

## MECHANISMS INVOLVED BEHIND THE GENERATION AND TRANSPORTATION OF SHORT-CHAIN FATTY ACIDS AND THEIR ROLE IN MODULATING PATHOPHYSIOLOGICAL CHANGES IN THE GUT

### Generation of short-chain fatty acids

**Figure 2:**
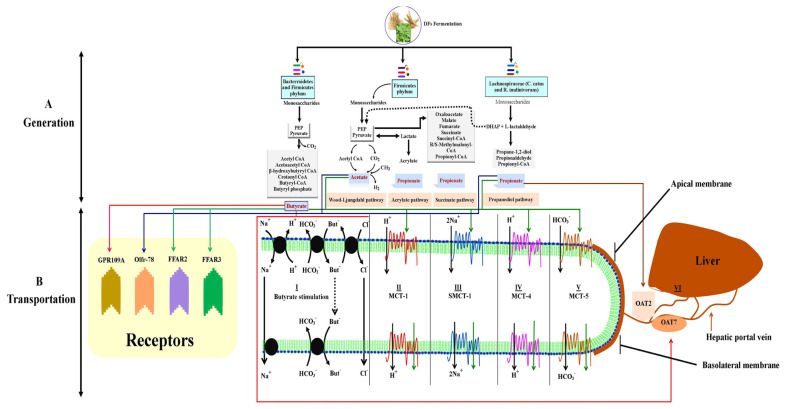
(A) Gut microbial fermentation and enzymatic pathways involved in acetate, propionate, and butyrate generation. Butyrate is produced either by the precipitation of two molecules of acetyl CoA or by enzyme butyryl- CoA:acetate-CoA-transferase. Acetate is produced either by acetyl CoA or by the Wood-Ljungdahl pathway. Propionate is formed through three pathways namely acrylate, succinate, and propanediol pathways. (B) The proposed transport mechanisms of short-chain fatty acids (SCFAs). I) butyrate stimulation of Na^+^ and Cl^−^, II) transportation of SCFAs via monocarboxylate transporter (MCT)-1, III) transportation by sodium-coupled monocarboxylate transporter-1, IV) SCFAs which are not absorbed by colonocytes transported through a basolateral membrane, where MCT-4 transports SCFA anions in an H^+^-dependent electroneutral manner, V) transportation by MCT-5 is via unknown HCO_3_^−^ exchanger, and VI) transportation of unabsorbed propionate and butyrate into the liver by organic anion transporter (OAT) 2 and 7 respectively via sinusoidal membrane of liver cells (hepatocytes).

Microbiota hydrolyze DFs into oligosaccharides and then produce monosaccharides in the hindgut under anaerobic environment conditions and produce SCFAs. This production of SCFAs consists of enzymatic pathways which are regulated by several bacterial species (Figure 2). The prime pathways for SCFAs generation are the Embden-Meyerhof-Parnas pathway (glycolytic pathway) and pentose phosphate pathway driven by *Bifidobacteria* which transform monosaccharides into phosphoenolpyruvate (PEP) [62]. The PEP is then converted into alcohols or organic acids (Figure 2A).

The enzymatic pathways for the synthesis of SCFAs have been previously described. In brief, oxygen-sensitive Wood-Ljungdahl pathway of SCFAs production was detected by radioisotope analysis and in this, propionate production was detected via CO_2_ fixation pathway and butyrate by the condensation of acetyl-S coenzyme A [63]. The main bacteria engaged in butyrate production were *Cytophaga* and *Flavobacterium* belonging to *Bacteroidetes phylum* (main bacterial spp; *Coprococcus* species, *Clostridium leptum*, *Faecalibacterium prausnitzii*, and *Roseburia* species belonging to both *Bacteroidetes* and *Firmicutes phyla*) [64] (Figure 2A). The Wood-Ljungdahl pathway is carried out by acetate-producing bacteria namely *Acetogens* (*Firmicutes phylum*) [65] (Figure 2A). Besides this, there are other pathways such as the fructose-6-phosphate phosphoketolase pathway present in the *Bifidobacterium* genus, also known as the *Bifidobacterium* pathway. In this pathway, the *Bifidobacterium* genus uses monosaccharides to produce SCFAs, particularly acetate. Propionate is produced by the acrylate pathway. Reichardt et al [66] described three pathways used by bacteria for the production of propionates such as succinate pathway, acrylate pathway, and propanediol pathway (Figure 2A). In these pathways, two species of *Lachnospiraceae* such as *C. catus* and *R. inulinivorans* have been seen to switch butyrate to propionate production from different substrates.

### Transportation of short-chain fatty acids

The epithelial cells of the colon i.e. colonocytes are mostly studied for the transportation of SCFAs (Figure 2B). The apical membrane gains the SCFAs in two ways i.e. active transport of dissociated SCFA anions, and passive diffusion of undissociated SCFAs. Three mechanisms support the transportation of SCFA anions via the apical membrane of colonocytes. In the first mechanism, the transporter introduces SCFA anions to HCO_3_^−^ to form SCFA-HCO_3_^−^ exchange in the vesicle and then secret it into the gut lumen. So this exchange is independent of Na^+^ transporter and Cl^−^-HCO_3_^−^ exchange [67]. The second mechanism involves the members of family of monocarboxylate transporters that increase the chemical reaction of SCFA anions with cations [68]. The monocarboxylate transporter-1 (MCT-1) transports SCFAs in an H^+^ dependent electroneutral manner in the apical membrane of enterocytes. Besides this, MCT-1 shows high expression in the lymphocytes which denotes its role in SCFA transportation. The third mechanism of SCFA anion transportation is carried by the sodium-coupled monocarboxylate transporter-1 (SMCT-1) [69]. In this, the SCFA anions are introduced to Na^+^ transporters in a 1:2 stoichiometry to increase water and Cl^−^ absorption (Figure 2B). The SMCT-1 is highly expressed in the large intestine, kidney, and thyroid gland, where it transports butyrate with high affinity as compared to propionate and acetate [70].

The leftover SCFAs, not absorbed by the colonocytes, are transported towards the basolateral membrane. This membrane contains MCT-4 and MCT-5 transporters. The MCT-4 transporter, transports SCFA anions in an H^+^-dependent electroneutral manner, however, MCT-5 transporter, transports SCFA through HCO_3_^−^ exchangers [71] (Figure 2B). The transporters of SCFAs have been observed from duodenum, ileum, ceca, colon, lungs, and liver, however, the transporters for the uptake of SCFAs from the blood are mostly undefined. Organic anion transporters such as organic anion transporter 2 and 7 are involved in the transportation of propionate and butyrate respectively through the sinusoidal membrane of liver cells (hepatocytes) [72]. In the liver, the propionate and acetate can be used as substrates for the energy-producing tricarboxylic acid cycle to produce glucose. For better understanding, the transportation and uptake mechanisms for SCFAs in various tissues should be investigated.

### Role of short-chain fatty acids in modulating the pathophysiological changes in the gut

It is known that SCFAs play a key role in modulating the gut health of pigs and poultry species (Table 2). SCFAs undergo antimicrobial pathways and enter the membrane of the pathogenic bacteria. The cytoplasmic pH of bacteria is generally neutral while SCFAs exist in associated or dissociated forms [73]. When SCFAs attack, they dissociate into protons and anions and reduce the pH of a bacterial cell, failing to maintain which, the bacterial cell gets destroyed. Antimicrobial activation of formate, acetate, and propionate has been documented in *in-vitro* studies. The *in-vivo* studies conducted in humans, pigs, and broilers have also shown antimicrobial effects of SCFAs on pathogens. In this regard, supplementation of piglets’ diet with wheat, improved the production of SCFAs and reduced the incidences/infection of *E. coli* [74]. Similarly, supplementation of butyralated high-amylose maize starch to broilers challenged with necrotic enteritis resulted in the production of ileal acetate and reduction in caecal pH which ameliorated the negative effects of disease [75]. In another study supplementation of wheat bran and aArabinoxylo-oligosaccharides in broilers promoted butyrate, propionate, and *Firmicutes* to *Proteobacteria* production and decreased caecal *Enterobacteriaceae* [76]. Moreover, the supplementation of chicory roots and sweet lupins increased the abundance of commensal microbiota i.e. *Bifidobacterium thermacidophilum* subspp. *Porcinum*, and *Megasphaera elsdenii*, lactate producers and lactate utilizing butyrate producers respectively and attenuated the abundances of intestinal spirochaete and *B. hyodysenteriae* [77]. So it is clear that fiber intake helps in improving gut health and decreases the load of pathogenic bacteria, however, it is still to find the best source or combination of fibers that can maintain gut health and eliminate chances of *E.coli*, *Salmonella*, and necrotic enteritis type of infections from poultry species. In addition to that how fiber sources decrease the involvement of pathogens and what are specific pathways involved, need further investigations.

## RECEPTORS, TARGET TISSUES, AND FUNCTIONS OF SHORT-CHAIN FATTY ACIDS

The SCFAs regulate the immune system through signaling mechanisms, the understanding of which may help improve immune system and reduce risk of certain diseases. There are two mechanisms by which SCFAs modulate immune cell chemotaxis, cytokines, and reactive oxygen species (ROS) of the host. The first mechanism is the activation of G-protein-coupled receptors (GPCRs) such as free fatty acid receptors (FFAR)-2 and -3 (also known as GPR43 and GPR41 receptors), niacin receptor 1, or GPR109A or hydroxyl-carboxylic acid 2 receptor, and olfactory receptor (Olfr78) [78,79]. Currently, we have reviewed four SCFA-sensing GPCRs which are expressed on IECs, endocrine cells, and leukocytes and play a central role in the regulation of metabolism (Table 3). The second mechanism consists of the direct inhibition of HDACs [80]. To get insight into HDACs inhibition, the mechanisms supporting SCFA-facilitated HDAC inhibition and their immunological consequences are discussed here.

### Pro- and anti-inflammatory effects of short-chain fatty acids in immune cells

**Figure 3:**
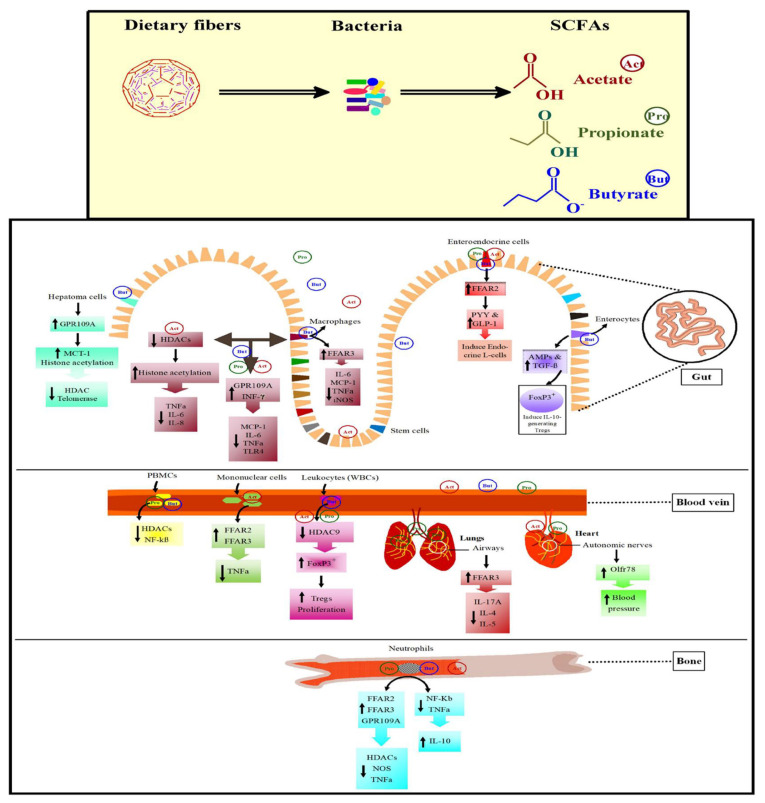
Synthesis of short-chain fatty acids (SCFAs) serves as an integrative bridge with intestinal epithelium by binding G-protein-coupled receptors (GPCRs) or inhibiting histone deacetylases (HDACs) mechanisms. Dietary fibers are converted into SCFAs such as acetate, propionate, and butyrate by gut microbiota. Then they go through biological processes and modulate pro- and anti-inflammatory immune phenotypes through activating GPCRs and blockading HDACs mechanisms in the gut, blood veins, and bodily tissues including the heart, lungs, and bones. Butyrate is one of the main SCFA that is metabolized by mature enterocytes by anaerobic β-oxidation and provides a maximum of the energy up to 60% to 70%. Butyrate increases the transforming growth factor-beta (*TGF-β*) and antimicrobial peptides expression in enterocytes [91]. The regulation of *TGF-β* increases the production of *IL-10*-generating Tregs in the colon. Acetate increase the expression of FFAR2 and FFAR3 which induce histone acetylation and cause inhibition of HDACs with decreased inflammatory cytokines production such as *TNFα*, *IL-6*, and *IL-8*. Propionate has been used for the treatment of lungs of allergic mice [83] which activates the expression of FFAR3 with reduced inflammatory mediators such as *IL-17A*, *IL-4*, and *IL-5*. FFAR, free fatty acid receptor; IL, interleukin.

The host immune system impedes the pathogens by producing inflammatory cytokines. Though, unnecessary secretion of cytokines gives rise to systemic inflammation. SCFAs modify systemic inflammation by modulating the release of immune cell cytokines, ROS, and chemotaxis (Figure 3). Propionate and butyrate activate FFAR2/3 or GPR109A or inhibit HDACs to decline the nitric oxide synthase and tumor necrosis factor-alpha (*TNFα*) expression in monocytes. Upon tracheal inflammation, SCFAs activate FFAR2 and FFAR3 from the macrophages and neutrophils which lead to the reduction of interleukin-8 (*IL-8*) in the trachea [81]. Butyrate acts anti-inflammatory factor in macrophages by activating FFAR3 and reducing interleukin-6 (*IL-6*), monocyte chemoattractant protein-1 (*MCP-1*), *TNFα*, and inducible nitric oxide synthase [82]. In mononuclear cells of humans and mice, acetate had anti-inflammatory effects and it inhibits lipopolysaccharides (LPS)-induced *TNFα* production through FFAR2 and FFAR3 activation. Treatment of allergic mice with propionate decreased inflammatory mediators, e.g. interleukin-17A (*IL-17A*), *IL-4*, and *IL-5* through FFAR3 activation pathway [83]. Similarly, propionate was found useful in treating lungs of allergic mice.

Activation of FFAR2 and FFAR3 down-regulate the expression of nuclear factor kappa B (*NF-KB*) downstream genes and are linked with the regulation of phosphoinositide 3-kinase, mitogen-activated protein kinases, c-Jun N-terminal kinase, extracellular signal-regulated kinase (*ERK*), p38 mitogen-activated protein kinase (*p38MAPK*), and rapamycin signaling pathways [84]. Upon activation of FFA receptors, acetate regulates *p38MAPK* and extracellular signal-regulated kinases 1/2 (*ERK1/2*) signaling pathways thus enhancing the synthesis of cytokines (chemokine (C-X-C motif) ligand1/2 and *IL-6*). In mice, the knockout of FFA receptors decreased *IL-6* synthesis and delay the expression of chemokines and interferon-gamma (*INF-γ*) [85]. These hallmark studies determine the pro-inflammatory effects of activated FFAR2 and FFAR3.

The expression of GPR109A in macrophages by enhanced *INF-γ* represents its importance in inflammation and immune regulation system. The activation and regulation of GPR109A blockade the production of *MCP-1*, *IL-6*, and *TNFα* and the expression of TLR4, thus, reducing the chances of atherosclerosis [86]. Butyrate on activating GPR109A acts as an anticancer mediator in human cancer cell lines. *In vitro* treatment of human hepatoma cells by butyrate decline the activity of telomerase through HDAC blockade. Butyrate as an activator of GPR109A in human colon cancer cells enhances the butyrate transporter MCT-1 expression and further increases apoptosis independent of HDAC inhibition [87]. This suggests that the activation of GPR109A may directly halt colon cancer development or indirectly transport the butyrate to the cell by increasing MCT-1 expression. The enhanced MCT-1 expression in the colon cancer cells is necessary for histone acetylation and also for its anti-tumorigenic function. The intake of whole grain and cereals were coupled with decreased incidence of colorectal cancer predicting the efficient role of SCFAs in cancer treatment [88].

Gut microbial fermentation of polysaccharides undergoes SCFAs production which goes through the portal vein circulation and becomes a part of plasma. A novel receptor of SCFAs “Olfr78” has been observed in blood vessels and autonomic nerves in heart [79] (Figure 3). In a study, antibiotics were used to reduce a load of gut microbiota to notice whether the antibiotics affect blood pressure (BP) in *Olfr78*^−/−^ mice or not. The results showed a significant increase in BP in Olfr78-deficient mice which suggests that propionate and acetate produced by gut microbiota might be involved in controlling BP. Based on current literature, we can say that SCFAs act as a bridge in maintaining gut health (Figure 3). In poultry species, the role of SCFAs receptors and their efficient participation in inhibiting HDACs has not yet been studied. So further research is required to unveil these processes in poultry.

### Short-chain fatty acids (SCFAs)-facilitated histone deacetylases (HDACs) inhibition

Many of the HDACs show expression on smooth vascular muscle cells, immune and endothelial cells. Microflora helps SCFA-facilitated HDAC inhibition and protects immune system. The exact mechanism supporting SCFA-facilitated HDAC inhibition is unclear however it is proposed that two mechanisms are involved i.e. the expression of SMCT-1 transporter and the activation of GPCRs. The SMCT-1 mechanism might cause direct inhibition of HDACs by entering into the cells with the help of transporters. The SMCT-1 promotes butyrate- and propionate-induced barrier of a murine dendritic cell which results in induced HDAC inhibition and DNA acetylation [89]. The GPCRs mechanism involves indirect inhibition of HDACs by SCFAs. For example, the stimulation of FFAR3 in Chinese hamster ovary cell lines inhibited HDACs and caused suppression of histone acetylation [90]. Not only FFAR3 but other receptors of SCFAs may involve in the inhibition of HDACs. SCFA-coupled HDAC inhibition that occurred in the colon was mostly dependent on FFAR2 [91]. Besides this, acetate may have controversial effects in stimulating inflammatory processes in a GPCRs-independent manner by regulating epigenetic modifications. Apart from this, propionate and butyrate may restrain the HDAC activity independent of FFAR3 and FFAR2 [82]. Further research is however required to find how SCFAs inhibit the activation of HDACs directly or indirectly in poultry species.

### Immunological consequences of short-chain fatty acids-induced histone deacetylases inhibition

Once SCFA-facilitated HDAC inhibition is developed, it results in a strong anti-inflammatory immune response as briefly described in Figure 3. Previously utilization of butyrate and propionate in the treatment of human peripheral blood mononuclear cells, reduced the production of LPS-induced-*TNFα* in a similar way to trichostatin A [92]. Similarly, the *in-vitro* use of acetate for the treatment of human macrophages remarkably diminished the HDAC activity and improved histone acetylation with reduced inflammatory cytokines production such as *TNFα*, *IL-6*, and *IL-8* [93]. It has been observed that *NF-kB* has a central role in the stimulation of inflammatory cytokines. This suggests that SCFAs might be involved in the modulation of *NF-kB* by HDAC inhibition. Similarly, treatment of neutrophils and LPS-activated mononuclear cells by butyrate and propionate decreased the *NF-kB* activity and *TNFα* production, and subsequently increased the production of anti-inflammatory cytokine interleukin-10 (*IL-10*) [94]. These results suggest the prominent role of SCFAs in the production of pro-inflammatory cytokines through inhibition of HDAC activity in humans and rodents. HDAC inhibition resulting from SCFAs is not limited to the cell’s innate immune system but it may also affect the white blood cells especially regulatory T cells (Tregs). The inhibition of HDAC9 in mice promotes the expression of forkhead box P3 (*Foxp3*) transcription factor, which increases proliferation of Tregs [95]. Similarly, butyrate increases the transforming growth factor-beta (*TGF-β*) and antimicrobial peptides expression in enterocytes [91]. The regulation of *TGF-β* increases the production of *IL-10*-generating Tregs in the colon, suggesting its role in restricting the proliferation of effector T cells. Though RNA-Seq analysis revealed that few birds including Falco cherrug, Falco peregrinus, and Parus humilis express *Foxp3* [96] yet it is not known whether the gut-derived microbial SCFAs disturb the Tregs in poultry because the *Foxp3* has not yet been identified in chickens and turkeys.

## CONCLUDING REMARKS AND FUTURE PERSPECTIVES

In conclusion, DFs can promote specific SCFA producing bacteria that may present a novel approach for managing *E. coli*, necrotic enteritis, and *Salmonella* in broilers and pigs. Fermentative bacteria mostly target the crop and cecum, whereas impacts of exogenously administered SCFAs may depend on the way of administration and hence diverse from microbially produced metabolites. As an example, oral administration of butyrate may target directly the small intestine and reach the periphery without being consumed by the colonocytes. Tissue-specific impacts of SCFAs have been revealed by propionate, whereby propionate-dependent gluconeogenesis improves metabolic health in the small intestine, whereas hepatic gluconeogenesis is injurious. Considering the expression of SCFA receptors in the blood vessels, small intestine, macrophages, and colonocytes, it can be vital to comprehend SCFA generation.

It is a major challenge to recognize the exact role of imposing opportunities for utilizing SCFAs in host pathophysiology which indispose a good understanding of the mechanisms through which SCFAs exploit their impacts in the animals’ gut, tissues, and organs. Of course, studies with the SCFAs seem to affect health via three main mechanisms; (I) activating GPCRs, (II) increasing histone acetylation and inhibiting HDAC activity, and (III) regulating anti-inflammatory mechanisms because of the first two mechanisms in the tissues and periphery, which can provide new and exciting possibilities for modulating poultry health.

To sum up, DFs can be considered key ancestral com pounds which regulate the macronutrients and preserve host physiology. In brief, we discussed how SCFAs are being generated, transported, and modulated the pro-and anti-inflammatory immune responses against pathogens and improve host physiology and gut health. Finally, screening novel fibers, both extracted and purified from food as well as those synthesized (prebiotics), and defining effective strategies to restore a high amount of fibers aiming at reintroducing the gut microbiome with important omitted SCFAs producing microbes, will be the next question to significantly affect gut microbiota-associated poultry diseases.

## Figures and Tables

**Table 1:** Sources, chemical composition, and mechanisms and action of dietary fibers

Dietary fiber	Chemical composition		Fermentation bacteria	Mechanisms and action	Sources

	Main chain	Branch chain			
Cellulose	β–(1–4)-D-glucose	-	*Alistipes* spp. and *Bacteriodetes* bacteria	The supplementation of cellulose changed the composition of microbiota at a level of phylum *Bacteroidetes* particularly the *Alistipes* genus in the cecum of broilers. This genus may improve the performance of broilers by producing succinate as an end product [18].	All plants, particularly cotton, some bacteria, and algae
Chitin	β–(1–4)-N-acetyl-D-glucose	-	*Oscillospira*	Ingestion of chitin (1.02 g/d) by hens throughout 21 weeks of age, enhanced the SCFA production and decreased the triglyceride content in serum and cholesterol content in serum and egg yolks [97].	Shells of crustaceans, insects, arthropods, yeast, and fungi
Chitosan	β-(1–4)-N-acetyl-D-glucosamine and β-(1–4)-D-glucosamine	-	*Bifidobacterium* spp. and *Lactobacillus* spp.	Chitosan oligosaccharides ingestion in mice increased SCFAs production that resulted in decreased *S. aureus*, *E.coli*, *non-typhoidal Salmonella*, *Listeria* spp., *Vibrio* spp., *B. Cereus*, and *Campylobacter* spp. [98].	The exoskeleton of crustaceans and cell walls of fungi but mostly produced by the deacetylation of chitin
Lignin	Polyphenols: p-coumaryl, coniferyl, sinapyl, syringyl, and guaiacyl alcohols	-	*Bifidobacteria*	Application of lignin in *in vitro* model has resulted in phenolic metabolites production along with stable *Bifidobacteria*. Furthermore, lignin improved the performance by reducing the colonization of *E. coli*, and Enterobacteriaceae in piglets [99].	Seeds (flax, pumpkin, sunflower, poppy, sesame), whole grains (rye, oats, barley), bran (wheat, oat, rye), beans, fruit (particularly berries), and vegetables
β-glucan	β-(1–3)-D-glucose and β-(1–4)-D-glucose	-	*Lactobacilli*, *Bifidobacteria*, *Roseburia hominis*, *Clostridiaceae* (*Clostridium orbiscindens* and *Clostridium* spp.), and *Ruminococcus* spp.	The intake of barley β-glucan in the human diet had resulted in a marked increase of *Clostridiaceae*, *Roseburia hominis*, and *Ruminococcus* spp. and SCFAs including butyric, acetic, propionic, and 2-methyl-propanoic acids [100].	Seaweed, brewer’s yeast, oats, lentinan (shiitake), barley, and maitake (grifola)
Hemicellulose					
Xyloglucan	β-(1–4)-D-glucose	Alfa-xylose attached at position 6- of β-D-glucose	*Clostridia*, *lactobacilli*, and *Bifidobacterium* (*endo-β-glucanase*)	The bacterial strains such as *Clostridia*, *lactobacilli*, and *Bifidobacterium* degraded the xyloglucan and generate acetate and propionate. The in vivo and in vitro application of xyloglucan restored the mucosal leakage by reducing the numbers of *E. coli* [101].	All vascular plants (i.e. gymnosperms, clumbosses, ferns, horsetails, and angiosperms) and seeds from tamarind and nasturtium.
Arabinoxylan	β-(1–4)-D-xylose	5-0-trans-feruloyl-α-(L-arabinose), L-Arabinose, 5-0-p-coumaroyl-α-Larabinose at position 2- or 3- of D-xylose	*Megamonas* and *Bifidobacterium* spp.	Arabinoxylan utilization by ducks had increased the stimulation of *Megamonas* and *Bifidobacterium* spp., which further resulted in increased concentration of branch-chain fatty acids (isobutyric acids) and SCFAs (butyrate, acetate, and propionate) [19].	ryegrass
Galactomannan	β-(1,4)-D-mannose	α-D-galactose at position 6- of β-D-mannose	*Lactobacillus* and *Bifidobacterium* spp.	The fermentation end products of prebiotic (B-galactomannan) are the acetates, butyrate, and propionates in humans. B-galactomannan ingestion enhanced mucus production which halted the access of *S. Enteritidis* to the epithelium in chickens [102].	Guar gum, locust bean gum, fenugreek, and alfalfa
Oligosaccharides					
Inulin	One terminal α-(1,2)-D-glucose and β-(2,1)-D-fructose	-	*Lactobacillus* and *Bifidobacterium* spp.	Inulin supplementation had been known to improve host gut inflammatory responses and gut health through promoting *Lactobacilli* and *Bifidobacteria* colonization and increasing SCFAs production largely butyrate, acetate, and propionate in broilers which exert antibacterial properties to combat *Salmonella* infection in chicks [15].	Rye, wheat, barley, onion, leek, garlic, and banana
Resistant starch	α-(1,4)-D-glucose	α-(1,4)-D-glucose and α-(1,6)-D-glucose	*Lactobacillus* spp.	The provision of resistant starch resulted in increased number of butyrate-producing *Bifidobacterium adolescentis*, *Parabacteroides distasonis*, *Faecalibacterium prausnitzii*, and total SCFAs in the lumen, small intestine, cecum, and colon of pigs [14].	Oatmeal, brown rice, corn, lentils, bananas, potatoes, yams, pasta, pearl barley, and navy beans
Galactooligosaccharides	One terminal β-(1,3)-D-glucose and β-(1,4)-D-galactose	-	*Bifidobacterium* and *Lactobacillus* spp.	*In vitro* studies demonstrated that Galactooligosaccharides supplementation increased acetate, lactate, and butyrate. Lactate and acetate formation is dependable on *lactobacillus* and *Bifidobacterium* fermentation. Its relative abundance within the intestinal tract has been observed to reduce the relative attack and adherence of *Salmonella* in chickens [103].	Milk, beans, root vegetables, etc.
Fructooligosaccharide	Derived from inulin hydrolysis: one terminal α-(1,2)-D-glucose and β-(2,1)-D-fructose,	-	*Bifidobacterium* and *Lactobacillus* spp.	The intake of dietary Fructooligosaccharide enhanced the production of SCFAs and reduced the colonization of *Salmonella* spp., *Clostridia perfringens*, and *E. coli* in broiler chickens [104].	Onion, chicory, garlic, asparagus, banana, artichoke, etc.
Gums					
Gum Guaran (Guar)	β-(1,4)-D-mannose	α-(1,6)-D-galactose	*Bacteroidetes*	*In vitro* application of gum, guar increased the production of SCFAs in the feces of mice which indicated reduced growth of *Desulfovibrio* [105].	Bean of guar plant, soy, wheat, corn, yeast, dairy, egg, gluten, and sugar
Gum Arabic	β-(1,3)-D-galactose	β-D-glucuronic acid, β-D-galactose, α-L-rhamnose, L-arabinose . Branches attached at position 6- of β-(1,3)-D-galactose	B*ifidobacterium* and *Lactobacillus* spp.	Gum Arabic fermentation in the large intestine of human resulted in increased *Bifidobacterium* spp. In a latest study, the *in vitro* application of gum Arabic has been observed to increase the production of SCFAs and antimicrobial activity against *clostridium* [106].	Acacia Senegal (Acacia)

**Table 2:** Role of short-chain fatty acids in modulating the pathophysiological changes in pigs and poultry species

Origin	Virulence	Virulent factor	Challenge	Dietary fibers	Pathophysiological effects	Reference
Broilers	Necrotic enteritis and inflammation of the small intestine	Lactose-negative *enterobacteria* and *clostridium perfringens*	S. Typhimurium DT110	Whole wheat and oat hulls	Increased hydrochloric acid secretion and grinding processes in the gizzard, and reduced its pH	[107]
Broilers	Necrotic enteritis and inflammation of the small intestine	C. perfringens	Necrotic Enteritis	The acetylated high amylose maize starch and Butyralated high amylose maize starch	Increased short-chain fatty acids (SCFAs) generation and decreased luminal pH	[75]
Broilers	Paratyphoid infections, *S*. Enteritidis, and foodborne diseases	*Salmonella enterica* and *E. coli*	Streptomycin resistant *S. enterica* serotype Enteritidis phage type 4 strain 147 (SE147)	Wheat bran and Arabinoxylo-oligosaccharides	Increased butyric acid, propionic acid, and *Firmicutes* to *Proteobacteria* production and decreased caecal *Enterobacteriaceae* levels	[76]
Broilers	Destroy the epithelial mucosal layers and infection of Peyer’s patches of the small intestinal wall	*S. Typhimurium*	**S. Typhimurium** invasion genes (invA, B, C, and D)	*Salmonella* with wheat bran	Decreased the impacts of hilA (a transcriptional activator of *Salmonella* pathogenicity island I vital for *Salmonella*) into epithelial cells	[108]
Laying hens	Destroy the epithelial mucosal layers and infection of Peyer’s patches of the small intestinal wall	*Salmonella*	Gavage	Fructooligosaccharide	Decreased the intestinal bacterial populations by increasing the growth of *Lactobacillus* and *Bifidobacterium* spp.	[109]
Piglets	Post-weaning diarrhea	*E. coli* and *Salmonella*	-	Oat hulls	Decreased fecal biogenic amines, cadaverine, and β-phenylethylamine.	[110]
Piglets	Post-weaning diarrhea	*E. coli*	*E. coli* K88	Wheat bran	Increased butyric acid and total SCFAs production with reduced intestinal enterobacterial populations particularly challenged Ileal *E. coli* K88 adhesions.	[74]
Piglets	Diarrhea	*E. coli*	*E. coli*	Inulin	Increased Lactobacillus: coliform ratio and SCFA concentrations.	[111]
Piglets	Intestinal mucosal damage and diarrhea	*E. coli*	*Enterotoxigenic E. coli*	10% Wheat bran fiber and pea fiber	Increase *Lactobacillus* in ileum and *Bifidobacterium* populations in colon which further increased colonic goblet cells, peptide trefoil factors, and villous height: crypt depth ratio in the ileum.	[112]
Pre-weaned pigs	*Salmonella* induced diarrhea	*S. typhimurium*	*S. typhimurium*798	Fermentable fiber	Increased SCFAs in the colon and glutamine transport.	[113]
Pigs	Swine dysentery (contagious diarrheal disease) and Trichuris suis (whipworm)	Intestinal spirochaete and *B. hyodysenteriae*	*B. hyodysenteriae*	Chicory root (fructans) and sweet lupins (galactans)	Increased the abundance of commensal microbiota such as *Bifidobacterium thermacidophilum* subsp. porcinum and *Megasphaera elsdenii*, lactate producers and lactate utilizing butyrate producers respectively.	[77]
Pigs	Swine dysentery (SD) (contagious diarrheal disease)	Affected large-intestinal microbiota to induce extensive inflammation and necrosis of the epithelial surface of the caecum and colon.	*B. hyodysenteriae*	Inulin and lupins	Increased the caecal SCFAs with reduced concentration of SD colonization	[114]

**Table 3:** Summary of the currently recognized SCFAs-activated GPCRs including their ligands, expression, and functions

Receptor	Ligands	Expression in tissues	Expression in cell types	Functions	References
GPR43 (FFAR2)	Acetate, propionate, butyrate, caproate, and valerate	Intestine, immune cells, murine hemopoietic tissues, and spleen	Endocrine L-cells, colonocytes, enterocytes, eosinophils, basophils, neutrophils, monocytes, dendritic cells, mucosal mast cells, and bone marrow	Anorexigenic effects through the secretion of peptide YY and glucagon-like peptide-1, development or differentiation of immune cells, anti-inflammatory role in reducing the risk of preterm labor induced by pathogens, decreases cyclic adenosine monophosphate (cAMP) levels and increases cytoplasmic calcium concentrations, inhibits NF-KB, and reduces the expression of pro-inflammatory cytokines, *IL-6* and *IL-1β*.	[78,115]
GPR41 (FFAR3)	Acetate, propionate, butyrate, caproate, and valerate	Adipose tissues, spleen, intestine, immune cells, and pancreas	Adipocytes, monocytes, enteroendocrine L-cells, neutrophils, monocyte-derived dendritic cells, and peripheral blood mononuclear cells	Inhibits adenylyl cyclase, reduces the levels of cAMP, and stimulates sympathetic activation by acting on the sympathetic ganglion.	[116]
GPR109A or HCA2 or NIACR1	Butyrate and niacin	Immune cells, intestine, and adipose tissues	Dermal dendritic cells, monocytes, macrophages, neutrophils, and adipocytes	Suppresses lipolysis and plasma-free fatty acid levels and regulates the vascular inflammation in atherosclerosis	[86]
Olfr78	Acetate and propionate	Kidney, colon, lungs, heart (autonomic nerves), and prostate	Juxtaglomerular cells, enteroendocrine cells, airway smooth muscle cells, prostate epithelium, and melanocytes	Mediates renin secretion in response to SCFAs and controls blood pressure system	[79,117]

SCFAs, short-chain fatty acids; GPCRs, G-protein-coupled receptors; IL, interleukin; GPR43, free fatty acid receptor; GPR109A, G-protein-coupled receptor 109A; HCA2, hydroxycarboxylic acid receptor 2; NIACR1, niacin receptor 1; Olfr78, olfactory receptor.
